# Targeting EGFR/HER2 pathways enhances the antiproliferative effect of gemcitabine in biliary tract and gallbladder carcinomas

**DOI:** 10.1186/1471-2407-10-631

**Published:** 2010-11-18

**Authors:** Ymera Pignochino, Ivana Sarotto, Caterina Peraldo-Neia, Junia Y Penachioni, Giuliana Cavalloni, Giorgia Migliardi, Laura Casorzo, Giovanna Chiorino, Mauro Risio, Alberto Bardelli, Massimo Aglietta, Francesco Leone

**Affiliations:** 1 Department of Medical Oncology, University of Torino Medical School, Institute for Cancer Research and Treatment, (str. Provinciale 142 km 3.95), Candiolo, (10060), Italy; 2 Unit of Pathology, Institute for Cancer Research and Treatment, (str. Provinciale 142 km 3.95), Candiolo, (10060), Italy; 3 Laboratory of Cancer Genomics, Fondazione Edo ed Elvo Tempia Valenta", (via Malta 3), Biella, (13900), Italy; 4 Laboratory of Molecular Genetics, The Oncogenomics Center, University of Torino Medical School, Institute for Cancer Research and Treatment, (str. Provinciale 142 km 3.95), Candiolo, (10060), Italy

## Abstract

**Background:**

Advanced biliary tract carcinomas (BTCs) have poor prognosis and limited therapeutic options. Therefore, it is crucial to combine standard therapies with molecular targeting. In this study EGFR, HER2, and their molecular transducers were analysed in terms of mutations, amplifications and over-expression in a BTC case series. Furthermore, we tested the efficacy of drugs targeting these molecules, as single agents or in combination with gemcitabine, the standard therapeutic agent against BTC.

**Methods:**

Immunohistochemistry, FISH and mutational analysis were performed on 49 BTC samples of intrahepatic (ICCs), extrahepatic (ECCs), and gallbladder (GBCs) origin. The effect on cell proliferation of different EGFR/HER2 pathway inhibitors as single agents or in combination with gemcitabine was investigated on BTC cell lines. Western blot analyses were performed to investigate molecular mechanisms of targeted drugs.

**Results:**

EGFR is expressed in 100% of ICCs, 52.6% of ECCs, and in 38.5% of GBCs. P-MAPK and p-Akt are highly expressed in ICCs (>58% of samples), and to a lower extent in ECCs and GBCs (<46%), indicating EGFR pathway activation. HER2 is overexpressed in 10% of GBCs (with genomic amplification), and 26.3% of ECCs (half of which has genomic amplification). EGFR or its signal transducers are mutated in 26.5% of cases: 4 samples bear mutations of PI3K (8.2%), 3 cases (6.1%) in K-RAS, 4 (8.2%) in B-RAF, and 2 cases (4.1%) in PTEN, but no loss of PTEN expression is detected. EGI-1 cell line is highly sensitive to gemcitabine, TFK1 and TGBC1-TKB cell lines are responsive and HuH28 cell line is resistant. In EGI-1 cells, combination with gefitinib further increases the antiproliferative effect of gemcitabine. In TFK1 and TGBC1-TKB cells, the efficacy of gemcitabine is increased with addiction of sorafenib and everolimus. In TGBC1-TKB cells, lapatinib also has a synergic effect with gemcitabine. HuH28 becomes responsive if treated in combination with erlotinib. Moreover, HuH28 cells are sensitive to lapatinib as a single agent. Molecular mechanisms were confirmed by western blot analysis.

**Conclusion:**

These data demonstrate that EGFR and HER2 pathways are suitable therapeutic targets for BTCs. The combination of gemcitabine with drugs targeting these pathways gives encouraging results and further clinical studies could be warranted.

## Background

Biliary tract carcinomas (BTCs) are rare primary malignancies originating from the epithelium of the biliary tree and lead to intrahepatic (ICCs), extrahepatic (ECCs), and gallbladder cancers (GBCs). Most patients are diagnosed when the disease is unresectable and survival is poor, with less than 5% of patients surviving beyond 5 years [1,2]. Chemotherapy has a limited impact on the natural history of the disease and several drugs or drug combinations have been tested with response rates ranging from 0% to 40%. Phase II studies have demonstrated that the best results were obtained with gemcitabine (Gem) reaching a 36% of response rate and 15.4 months of median survival [3]. More recently a multicenter, randomized phase III trial (the UK ABC-02 trial) recruiting 410 patients with advanced BTCs demonstrated that the median progression free survival was greater with the association of Gem with cisplatin than Gem alone (8 vs. 5 months) [4].

Effective therapeutic agents based on a better comprehension of cellular and molecular pathogenesis of BTCs are required. Preclinical studies suggest that the Epidermal Growth Factor Receptor (EGFR), HER2, and their pathways have a crucial role in tumor growth [5]. The EGFR/HER2 signaling pathway exerts its biological effects via multiple signaling cascades including phospholipase C, Ca2+/calmodulin-dependent kinase (CaMK/PKC), Ras/Raf/Mitogen/Activated Proteine Kinases (MAPK), the phosphatidylinositol 3'-kinase (PI3K)/Akt/mammalian target of rapamycin (mTOR), PI3K/Akt/GSK, and Janus-associated kinase (JAK)/signal transducer and activator of transcription protein (STATs) [6-8].

In addition, EGFR signaling regulates the synthesis and secretion of several different angiogenic growth factors in tumor cells, including vascular endothelial growth factor (VEGF), interleukin-8 (IL-8), and basic fibroblast growth factor (bFGF) [9].

In cholangiocarcinoma, as well as in normal cholangiocytes, bile acids activate the two main signaling pathways (Ras/Raf/MAPK and the PI3K/Akt/mTOR) via a TGF-α-dependent mechanism. Bile acid mitogenesis may facilitate the progression of cholangiocarcinoma and blocking the TGF-α/EGFR autocrine pathway attenuates bile acid-stimulated growth of cholangiocarcinoma cell lines [10-12]. On these bases, several lines of evidence may point to the usefulness of EGFR targeting as an adjuvant therapy in cholangiocarcinoma. We previously reported that 15% of biliary tree and gallbladder carcinomas had EGFR gene mutations in the tyrosine kinase (TK) domain and that the mutations led to activation of one or both of the EGFR signal transduction pathways [13]. Some of these mutations are identical to those previously reported to confer sensitivity to some TK inhibitors like erlotinib and gefitinib in non small cell lung cancer (NSCLC) [14]. However, these inhibitors are ineffective if used in the presence of mutations in EGFR downstream transducers, such as K-RAS, B-RAF, PI3K or phosphatase and tensin homolog deleted on chromosome 10 (PTEN) [15]. In NSCLC, increased copy number of the HER2 gene is associated with gefitinib sensitivity in EGFR-positive patients, thus supporting the use of HER2 FISH analysis for selection of patients for TK inhibitor (TKI) therapies [16].

Somatic mutations in the PI3K gene have been frequently identified in colon and gastric carcinoma, and glioblastoma, but rarely in other cancers [17]. Functional analyses have revealed that these mutations increased kinase activity and induced transformation. In addition, in vitro experiments have demonstrated that PI3K oncogenic mutations promote sustained PI3K signaling, conferring resistance to gefitinib-induced apoptosis [18]. The tumor suppressor gene PTEN, that counteracts the activity of PI3K, was frequently mutated in high-grade glioblastoma, melanoma, prostate, and endometrium cancers [19]. These mutations caused loss of PTEN expression, constitutive activation of Akt, and resistance to gefitinib [20]. In vitro models demonstrated that the re-establishment of PTEN expression restores sensitivity to gefitinib [21]. All these data have derived from in vitro studies or from different series of patients in which only single aspects are studied therefore not allowing for evaluation of these findings as a whole. Due to prior experience with anti-EGFR treatment in lung and colorectal cancer patients, it has become clear that only a minority of patients with specific molecular abnormalities can benefit from these therapies. Philip and coworkers reported some clinical activity of erlotinib as a single agent in cholangiocarcinoma, showing that 17% of patients were progression free after 24 weeks of treatment [22]. However, the lack of immunohistochemical and molecular studies did not allow the determination of which subgroups of patients would benefit most from these treatments. Strategies based on EGFR pathway targeting showed promising results [23-28].

Based on these premises, we decided that a careful investigation of EGFR- and HER2-related pathways in BTCs should be preliminary for clinical studies with targeted molecules, facilitating a guide to monitor parameters that are predictive of response. Therefore, the objectives of the current study were to investigate EGFR and HER2 pathway expression and activation in histological sections from patients and to evaluate the in vitro efficacy of selective inhibitors of these pathways as single agents or in combination with gemcitabine in BTC cell lines.

## Methods

### Patients and tissues

The study was conducted on archival formalin-fixed tissues derived from 49 Italian patients with BTCs diagnosed at the Institute for Cancer Research and Treatment (IRCC) from 2002 to 2005. Tumor specimens were obtained, along with prior informed consent, before any systemic treatment. Approval of ethical committee was not needed for this study.

Histological type was determined according to World Health Organization criteria and tumor stage at the time of diagnosis was determined according to TNM classification system [29,30].

Patient characteristics, including sex, age, tumor origin, stage and histological grading are summarised in table 1.

**Table 1:** Clinicalpathological parameters of BTC case series

Sample	Sex	Age	Origin	TNM *	Grade
1	M	75	GBC	T3N0M0	2

2	M	72	ICC	T3N1M0	3

3	F	48	ICC	T3N0M0	3

4	M	46	ECC	T3N1M0	3

5	M	70	ECC	T3N1M0	3

6	M	74	ECC	T3N1M0	3

7	M	69	ICC	TxN1M1	3

8	M	47	ICC	TxN1M1	2

9	F	50	ICC	T1N0M0	2

10	M	67	ICC	T3N0M0	2

11	M	50	ICC	T2NxM0	3

12	F	54	GBC	TxN1M1	2

13	M	73	ECC	T3N0M0	3

14	M	64	ECC	T3N0M0	2

15	M	53	ECC	T3N0M0	2

16	M	63	ECC	T4N0M0	2

17	F	57	ECC	T3N2M0	3

18	M	74	ECC	T2N1M0	3

19	M	69	ECC	T2N0M0	1

20	M	68	GBC	T2N0M0	2

21	M	66	ICC	T3N1M0	3

22	M	64	ECC	T2N0M0	3

23	F	75	GBC	T3NxM0	3

24	M	72	ECC	T3N1M0	2

25	F	66	ICC	T4N1M0	3

26	F	70	ECC	TxN1M0	1

27	F	71	GBC	T2N1M0	3

28	F	78	GBC	T2NxM0	3

29	F	60	ECC	T3N1M0	3

30	F	51	GBC	T3N1M0	2

31	F	41	ICC	NA	3

32	F	69	GBC	T2N1M0	3

33	F	84	GBC	T2NxM0	3

34	M	83	GBC	T2NxM0	3

35	M	56	GBC	T2NxM0	3

36	F	61	ICC	TxNxM1	3

37	F	72	ICC	TxNxM0	3

38	F	69	GBC	T3N0M0	3

39	M	52	ICC	TxNxM0	3

40	M	67	ECC	TxNxM0	2

41	M	66	ICC	T3NxM0	3

42	F	53	ECC	T3N1M0	2

43	M	68	ECC	TxNxM0	3

44	F	73	ECC	T2N0M0	2

45	F	55	GBC	T4N1M0	3

46	M	53	ICC	TxNxM1	3

47	M	66	ICC	T3NxM0	3

48	F	53	ICC	TxNxM0	3

49	M	67	ECC	T4N1M0	2

ICC: Intrahepatic cholangiocarcinoma; ECC: Extrahepatic cholangiocarcinoma; GBC: Gallbladder carcinoma, * TNM staging (see ref. [30])

### Drugs

Erlotinib and gefitinib were gifts from Roche and Astrazeneca respectively. They are reversible selective inhibitors of the tyrosine kinase domain of the epidermal growth factor receptor (EGFR). They act competitively at the ATP-binding site of the EGFR in order to inhibit ligand-induced tyrosine phosphorylation, thereby blocking ligand-induced activation of the receptor and downstream pathways.

Lapatinib, sorafenib, and everolimus were purchased by Sequoia (Sequoia Research Products Pangbourne, UK).

Lapatinib inhibits receptor signal processes by binding to the ATP-binding pocket of the EGFR/HER2 protein kinase domain, preventing self-phosphorylation and subsequent activation of the signal mechanism.

Sorafenib is a multikinase inhibitor that blocks tumor cell proliferation and angiogenesis by inhibiting serine/threonine kinases (c-RAF, mutant and wild-type B-RAF) as well as the receptor tyrosine kinases vascular endothelial growth factor receptor 2 (VEGFR2), VEGFR3, platelet-derived growth factor receptor (PDGFR), FLT3, Ret, and c-KIT. It has also been reported that sorafenib induces apoptosis through the inhibition of the translation and down-regulation of myeloid cell leukemia-1 (Mcl-1), a Bcl-2 family member.

Everolimus is a signal transduction inhibitor targeting mTOR (mammalian target of rapamycin)

All drugs were dissolved in DMSO (Sigma-Aldrich, St. Louis, MO, USA). Gemcitabine (GEMZAR) was from Lilly USA, and is a nucleoside analog that interferes with DNA replication.

### Cell lines and treatments

Four human cell lines of different histotype were used: two ECC cell lines- TFK1 and EGI-1 (WT and mutated on K-RAS respectively) kindly provided by Scherubl from the Institute of Physiology, Charité-Universitätsmedizin Berlin, Germany; one ICC cell line- HuH28 (mutated on PI3K); and one GBC cell line- TGBC1-TKB (deleted on PTEN) obtained from Cell Bank, RIKEN BioResource Center, Tsukuba, Japan. All cells were cultured in RPMI 1640 +10% FBS and 100 U/ml of penicilline/streptomicine. Cells were plated in 96 multiwell plates in a concentration of 3000 cells/well and incubated with 1:5 scalar doses of drugs (from 10 μM to 16 nM) for 72 hours. For combination experiments, drugs were administered concomitantly. Proliferation assays were performed by Cell Titer Glo Luminescent Cell Viability Assay and acquired by DTX880 (Beckman Coulter Inc). Drug interaction was assessed at a fixed 1:1 concentration ratio of gemcitabine and gefitinib/erlotinib/sorafenib/lapatinib/everolimus. The median Dose (Dm) inhibiting 50% of cell proliferation and its 95% confidence interval, isobologram analysis and dose-effect curves were calculated by CalcuSyn software (Biosoft, Cambridge, UK) based on Chou - Talalay method. The general equation for the classic isobologram is given by: combination index CI = (D)_1_/(Dx)_1 _+ (D)_2_/(Dx)_2 _where (Dx)1 and (Dx)2 in the denominators are the concentrations for D1 (gemcitabine) and D2 (targeted drug) alone that give x% inhibition, whereas (D)1 and (D)2 in the nominators are the doses of gemcitabine and targeted drug in combination that also induce the same effect (inhibit x%). CI<1, CI = 1 and CI > 1 indicated synergistic, additive and antagonistic effects, respectively. Cell lines showing Dm>10 μM were considered resistant to drug treatments. All tests were performed in quadruplicate and repeated in three independent experiments.

### Mutational analysis

Genomic DNA was extracted with the QIAamp DNA Mini Kit (Qiagen, Milan, Italy) following the manufacturer's instructions. Tumor portion and surrounding normal tissues were obtained by laser microdissector (VSL-337ND-S, Spectra-Physics, Mountain View, CA). Table 2 shows exons amplified by PCR with relative specific primers. The PCR products were purified using QIAquick PCR purification kit (Qiagen Milan, Italy) and sense and antisense sequences were obtained by using forward and reverse primers respectively. Each exon was sequenced using the BigDye Terminator Cycle sequence following the PE Applied Biosystem strategy and Applied Biosystems ABI PRISM3100 DNA Sequencer (Applied Biosystem, Forster City, CA). All mutations were confirmed by two independent PCR experiments and their somatic origin was demonstrated whenever possible excluding the presence of the same mutation in surrounding normal tissues.

**Table 2:** Sequences of forward and reverse primers (5'-3') used for PCR and sequencing

Gene	Exon	Forward primer	Reverse primer
EGFR	18	TCAGAGCCTGTGTTTCTACCAA	TGGTCTCACAGGACCACTGATT

EGFR	19	AAATAATCAGTGTGATTCGTGGAG	GAGGCCAGTGCTGTCTCTAAGG

EGFR	20	ACTTCACAGCCCTGCCGTAAAC	ATGGGACAGGCACTGATTTGT

EGFR	21	GCAGCGGGTTACATCTTCTTTC	CAGCTCTGGCTCACACTACCAG

PIK3CA	9	GGGAAAAATATGACAAAGAAAGC	CTGAGATCAGCCAAATTCAGTT

PIK3CA	20	CTCAATGATGCTTGGCTCTG	TGGAATCCAGAGTGAGCTTTC

KRAS	1	GGTGGAGTATTTGATAGTGTATTAACC	AGAATGGTCCTGCACCAGTAA

BRAF	15	TGCTTGCTCTGATAGGAAAATG	AGCATCTCAGGGCCAAAAAT

PTEN	5	GCAACATTTCTAAAGTTACCTA	CTGTTTTCCAATAAATTCTCA

PTEN	6	CATAGCAATTTAGTGAAATAACT	GATATGGTTAAGAAAACTGTTC

PTEN	7	CAGTTAAAGGCATTTCCTGTG	GGATATTTCTCCCAATGAAAG

PTEN	8	CTCAGATTGCCTTATAATAGTC	AACTTGTCAAGCAAGTTCTTC

PTEN	9	GTTCATCTGCAAAATGGA	GGTAATCTGACACAATGTCCTA

HER2	18	GTGAAGTCCTCCCAGCCCGC	CTCCCATCAGAACTGCCGACC

HER2	19	TGGAGGACAAGTAATGATCTCCTGG	AGACCAGAGCCCAGACCTG

HER2	20	GCCATGGCTGTGGTTTGTGATGG	ATCCTAGCCCCTTGTGGACATAGG

HER2	21	GGACTCTTGCTGGGCATGTGG	CCACTCAGAGTTCTCCCATGG

HER2	22	CCATGGGAGAACTCTGAGTGG	TCCCTTCACATGCTGAGGTGG

HER2	23	AGACTCCTGAGCAGAACCTCTG	AGCCAGCACAGCTCAGCCAC

### Immunohistochemistry

EGFR and HER2 expression was evaluated using PharmaDX (DakoCytomation, Carpenteria, CA) and HercepTest (Dako) kits respectively following manufacturer's instructions. EGFR intensity was scored from 1+ to 3+ and the threshold for positivity was 1+ staining intensity in 1% of tumor cells. HER2 staining was scored from 0 to 3+ using the scoring system outlined in the Dako HercepTest.

The anti-human TGF-α monoclonal antibody (clone 213-4,4; Oncogene Research Products, Cambridge, MA) was used for TGF-α expression established semiquantitatively from the percentage of TGF-α^+ ^cells and the staining intensity. Tumors were graded as negative (0 to less than 10% positive cells), + (≥10% to < 25% positive cells), ++ (>25% to < 50% positive cells) and +++ (> 50% positive cells). The percentage of immunopositive cells was calculated by counting at least 1,000 cancer cells in contiguous fields with the greatest immunopositivity.

For EGFR downstream signaling detection, rabbit polyclonal antibodies anti-p-MAPK/Thr 202/Thr 204 and anti-p-Akt/Ser 473 (all from Cell Signaling Technology) were used. Positive immunostaining was scored + when present in more than 40% of cells and attributed to nuclear staining for p-MAPK and cytoplasmatic with a faint membranous staining for p-Akt.

PTEN expression, detected with the primary antibody anti-PTEN (clone 28H6, NeoMarkers, Fremont, CA 94539TS 106) was quantified by using a visual grading system based on the intensity of staining and the percentage of positive nuclei calculated counting of 1000 cells in 3 different optical fields at 40× magnification. PTEN immunostaining was scored into four groups from 0 to 3 as TGF-α scoring.

### Western blot analysis

Cells were lysed with boiling buffer (SDS 2.5%, TRIS HCl 0.125 M pH 6.8), scraped, boiled for 5 minutes at 100°C and centrifuged at 14,000 rpm for 30 minutes; 40 μg of proteins were electrophoresed on SDS-PAGE and transferred to 0.45-μm PVDF membranes (GE Healthcare). Nonspecific sites were blocked with 5% non-fat dry milk (BioRad Laboratories, Munchen, Germany) and membranes were immunoblotted with specific primary antibodies overnight followed by 1 μg/mL horseradish peroxidase conjugated secondary antibody. Antibodies against Akt, phosphorylated-Erk1/2 (Thr202/Tyr204), phosphorylated-Akt (Ser473), phosphorylated mTOR (Ser 2448), mTOR, anti-mouse and anti-rabbit antibodies linked with horseradish peroxidase were from Cell Signalling Technology (Beverly, USA); antibodies for Erk1/2, HER2, EGFR were from Santa Cruz Biotechnology; anti-PTEN and β-ACTIN were from MILLIPORE (Temecula, CA); antibody anti-vinculin was from Sigma (Sigma-Aldrich, St. Louis, MO, USA).

### HER2 Fluorescence *In Situ *Hybridization

Tissue sections (4-6 μm) were placed on silane-coated slides, deparaffinised, dehydrated, enzymatically digested with a commercial kit (Vysis, Downers Grove, IL, USA) and denaturated at 75°C for 5 minutes. Spectrum-Orange-labeled HER-2 and Spectrum-Green-labeled centromere 17 references (PathVysion™, Vysis-Abbott) were denaturated for 5' at 75°C and were applied to each slide. Slides were incubated for 5' at 79°C for codenaturation and placed in a humidified chamber at 37°C overnight for the hybridisation step. After washing, chromatin was counterstained with DAPI II (Vysis, Downers Grove, IL, USA). An average of 40 nuclei were analysed at five different target areas using H&E-stained sections as histotopographic reference. Our criteria for HER2 gene amplification were a HER2/centromere 17 ratio ≥2. Chromosome 17 numerical status was referred as polysomic when multiple 17 centromeric signals were present in >20% of the cancer cell population.

### Statistical Analysis

The R statistical language adaptation of Fisher exact test for non 2 × 2 contingency tables was performed in order to detect any significant association between histotype and the categorical variables describing the presence of mutations in EGFR or in specific signal transducers, the expression of EGFR, HER2, TGF-α, p-MAPK, p-Akt and PTEN. Mean quantitative values were compared using Student's t-test. The significance of difference between groups was assessed by a Pearson correlation analysis. A *P *value of less than 0.05 was considered significant. All *P *values were two-tailed.

## Results

### Expression of EGFR/HER2 proteins and related transducers in biliary tumors

**Figure 1:**
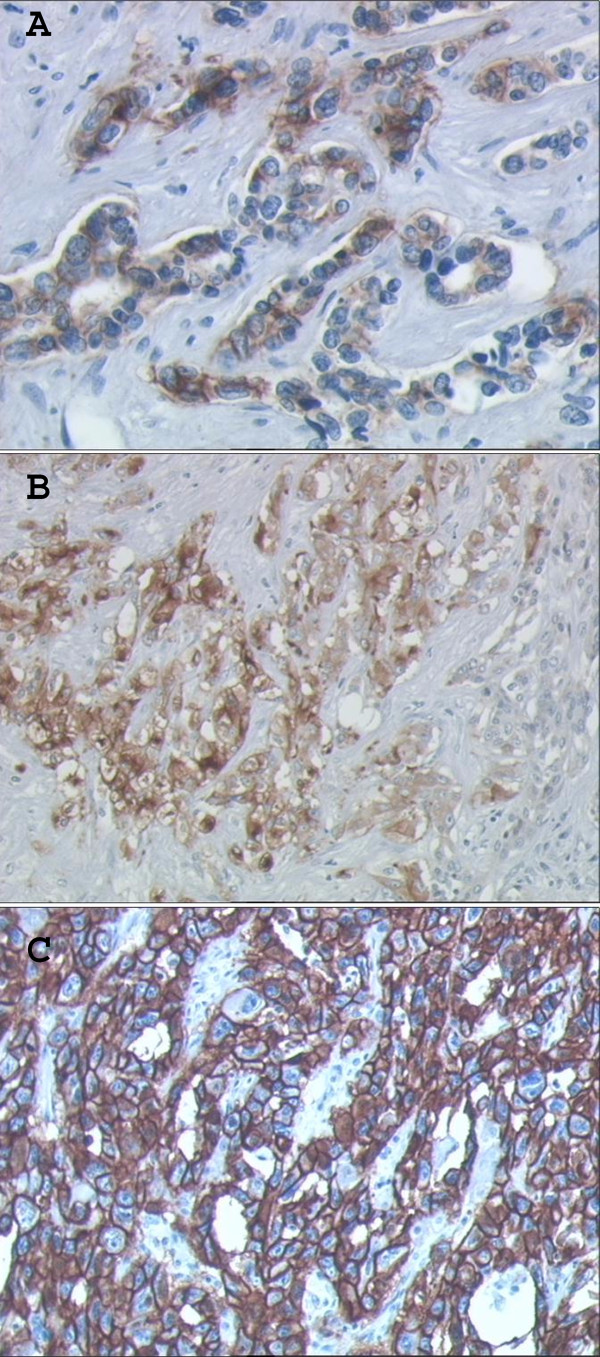
**Pattern of EGFR expression in the biliary tumors**. EGFR expression as typical cell membrane immunostaining: weak (1+ score; A); moderate (2+ score, B) and strong (3+ score, C). Magnification X400.

Immunoreactivity for EGFR was detected in all normal cholangiocyte and hepatocyte membranes. EGFR expression was present in all 17 ICCs: with an intensity of 3+ in 13/17 (76.5%), and 2+ in 3/17 (17.6%). One ICC with neuroendocrine differentiation was scored 1+ (5.9%). In the 19 ECCs, the expression pattern was more heterogeneous with 10/19 (52.6%) EGFR^+ ^cases: only 5/19 (26.3%) were scored 3+, 3/19 (15.8%) 2+, 2/19 (10.5%) 1+ and 9/19 (47.4%) were negative. In GBCs 5/13 (38.5%) expressed EGFR; 4/13 (30.8%) were scored 3+, 1/13 (7.7%) was 1+ and 8/13 (61.5%) were negatives. EGFR^+ ^cancers were significantly more frequent in ICCs than in ECCs or GBCs (table 3). No correlation was found between EGFR expression and histological grading in the different BTC subgroups. Figure 1 shows representative EGFR immunostaining.

**Table 3:** Expression of biomarkers and HER2 gene status in BTC samples from patients.

Sample	EGFR	TGF-α	P-MAPK	P-AKT	PTEN	HER2	HER2 FISH *
1	0	0	-	-	1+	0	ND

2	3+	+	-	-	1+	0	ND

3	3+	+	-	-	1+	0	ND

4	3+	+	-	+	3+	0	ND

5	3+	+	-	-	ND	1+	ND

6	3+	+	-	-	1+	0	ND

7	3+	+	-	+	2+	0	ND

8	3+	+++	-	+	3+	0	ND

9	3+	0	-	-	1+	0	ND

10	3+	0	+	+	2+	0	ND

11	3+	+++	+	+	3+	1+	ND

12	0	0	-	-	ND	ND	ND

13	2+	+	+	+	2+	0	ND

14	3+	+	+	-	2+	3+	RATIO: 10

15	2+	+	-	-	1+	2+	1.4 POLYSOMY 17

16	1+	0	-	-	1+	0	ND

17	0	0	-	-	1+	2+	1.7 POLYSOMY 17

18	2+	+	-	+	1+	0	ND

19	0	0	+	+	ND	2+	RATIO: 5.9

20	0	0	ND	ND	3+	0	ND

21	1+	++	+	+	3+	ND	ND

22	0	0	-	-	3+	0	ND

23	0	0	-	+	3+	0	ND

24	1+	+++	+	+	3+	0	ND

25	3+	+	+	+	1+	0	ND

26	0	0	-	+	3+	0	ND

27	0	0	+	-	3+	ND	ND

28	0	0	-	-	3+	0	ND

29	0	+	+	-	2+	0	ND

30	0	+	-	+	1+	3+	RATIO: 6.9

31	3+	0	+	+	3+	ND	ND

32	0	ND	+	-	2+	0	ND

33	3+	0	+	+	3+	0	ND

34	3+	+	-	+	3+	0	ND

35	3+	+	+	+	2+	ND	ND

36	3+	+	+	+	3+	ND	ND

37	3+	+++	+	+	2+	ND	ND

38	1+	++	ND	ND	2+	0	ND

39	3+	+	+	+	ND	0	ND

40	0	0	+	+	ND	0	ND

41	2+	+	ND	ND	2+	ND	ND

42	3+	+	ND	ND	3+	0	ND

43	0	0	-	-	3+	0	ND

44	0	0	+	-	2+	0	ND

45	3+	+	+	+	1+	0	ND

46	3+	+	-	+	3+	0	ND

47	2+	++	+	+	1+	ND	ND

48	2+	+	+	+	1+	ND	ND

49	0	0	-	-	3+	0	ND

ND: not determined,* HER2 FISH analysed as described in the "methods" section

Werneburg et al [10] demonstrated that EGFR was activated by bile acids in a TGF-α-dependent manner. On this basis, we decided to investigate if a pathological upregulation of this ligand might occur in cholangiocarcinoma cells. The expression of this ligand was analysed by immunohistochemistry in 49 BTC samples from patients.

Twenty nine out of 49 BTC (59.2%) resulted positive for TGF-α expression; in particular 14 out of 17 ICCs (82.4%), 10 out of 19 (52.6%) ECCs and 5 out of 13 (38.5%) GBCs were TGF-α^+^. Twenty seven out of 49 (55.1%) carcinomas displayed positive immunostaining for both TGF-α and EGFR. There was a significant relationship between EGFR and TGF-α expression in BTCs (p < 0.001).

**Figure 2:**
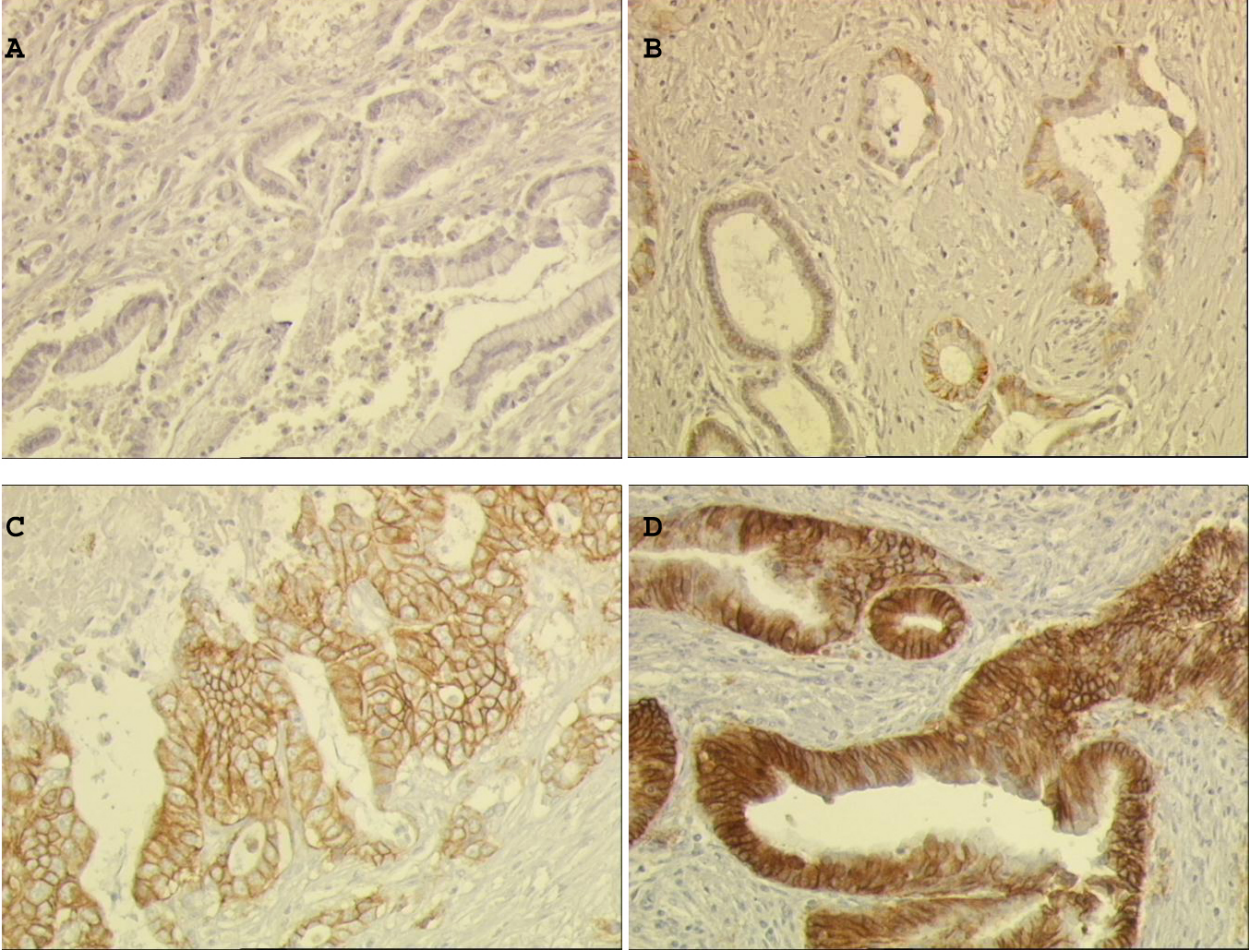
**Pattern of ErbB2 expression by HercepTest in the biliary tumors**. Scoring from 0 to 3+ (A, B, negative; C 2+ and D 3+). Magnification X200.

HER2 expression was performed in 10 ICCs, 19 ECCs and 10 GBCs, according to sample availability. Membranous expression was present in cancer cells, while normal cholangiocytes and stromal cells were negative. Seven of the 39 cases (17.9%) were HER2+; in particular 1/10 (10%) of ICC was scored 1+ and 1/10 (10%) GBC was 3+. Positive immunostaining for HER2 was detected in 5/19 (26.3%) ECCs. Figure 2 shows representative HER2 expression on BTC samples by HercepTest.

Phosphorylation status of downstream transducers MAPK (Erk1/2) and Akt was analysed by immunohistochemistry in all 49 BTCs. As shown in table 3, 10/17 ICCs (58.8%) presented p-MAPK and 13/17 (76.5%) were positive for p-Akt; co-expression of the two phosphorylated signaling proteins were detected in 10/17 (58.8%). On the contrary, in ECCs the p-MAPK or p-Akt were only detected in 7/19 (36.8%) with co-expression in 4/19 (21%). In GBCs the pattern of activated proteins was similar to that of ECC: 5/13 (38.5%) and 6/13 (46.1%) showed p-MAPK and p-Akt expression respectively, while the co-activation was found in 3/13 (23.1%). p-MAPK and p-Akt expression were higher in the ICCs compared to ECCs and GBCs (p < 0.05).

PTEN expression was observed in all BTCs and in normal cholangiocytes. Cancer cells showed a moderate or strong immunostaining, while normal cells presented weak immunostaining (score 2+ or 3+ *vs *1+).

### HER2 gene amplification

To determine if overexpression of HER2 protein is attributable to gene amplification, FISH analysis was performed on samples scored 2+ and 3+ by HercepTest. The two samples scored 3+ by HercepTest presented HER2 gene amplification. In particular HER2/centromere 17 ratio were 10 and 6.9, respectively. One out of 3 specimens overexpressing HER2 (scored 2+ by HercepTest) showed HER2 gene amplification with a ratio of 5.9. The remaining samples scored 2+ presented multiple 17 centromeric signals in more than 20% of tumor cells. The results of FISH analysis were shown in table 3.

### Mutational analysis

The mutational analysis of exons 18 to 21 of EGFR in this series has been published in a previous work [13] and these results, together with nine additional cases, are summarised in table 4. In this study, we also performed a systematic mutational analysis of HER2, PI3K, K-RAS, B-RAF and PTEN signal transducers. Sample number 39 showed a novel EGFR mutation (TGG to TAG) consistent with a nonsense substitution (W817Stop), leading to the production of a truncated protein lacking the intracytoplasmatic tail, which contains the interaction sites with the signal transducer PI3K.

**Table 4:** Mutations of EGFR, HER2 and their transducers found in samples from patients.

Sample	EGFR	KRAS exon 2	PI3K exon 9	PI3K exon 20	BRAF exon 15	PTEN
1	WT	WT	WT	WT	WT	WT

2	WT	WT	WT	WT	WT	WT

3	WT	WT	WT	WT	WT	WT

4	WT	WT	WT	WT	WT	WT

5	WT	WT	WT	WT	WT	WT

6	WT	WT	WT	WT	WT	WT

7	WT	WT	WT	WT	WT	WT

8	WT	WT	WT	WT	WT	WT

9	WT	WT	WT	WT	WT	WT

10	WT	WT	WT	WT	WT	WT

11	WT	WT	WT	WT	WT	WT

12	WT	WT	WT	WT	WT	WT

13	WT	WT	WT	WT	WT	WT

14	WT	WT	WT	WT	WT	WT

15	WT	WT	WT	WT	WT	WT

16	WT	WT	WT	WT	WT	WT

17	WT	WT	WT	WT	WT	WT

18	WT	WT	WT	WT	WT	WT

19	WT	WT	WT	WT	WT	WT

20	WT	WT	WT	WT	WT	WT

21	**K757R**	WT	WT	WT	WT	WT

22	WT	**G13D**	WT	WT	**V600E**	WT

23	WT	**G13D**	**E545K**	**H1047R**	WT	**T202I/E235G**

24	WT	WT	WT	WT	WT	WT

25	WT	WT	WT	WT	**V600E**	WT

26	**C775Y**	WT	WT	WT	WT	WT

27	WT	WT	WT	WT	WT	WT

28	WT	WT	WT	WT	WT	WT

29	**V843I**	WT	**Q546L**	WT	WT	**F271L**

30	WT	WT	WT	WT	WT	WT

31	**E872K**	WT	**E545A**	WT	**V600E**	WT

32	WT	WT	WT	WT	WT	WT

33	WT	WT	WT	WT	WT	WT

34	WT	WT	WT	WT	WT	WT

35	WT	WT	WT	WT	WT	WT

36	**T790M**	WT	WT	WT	WT	WT

37	WT	WT	WT	WT	WT	WT

38	WT	WT	WT	WT	WT	WT

39	**W817STOP**	WT	WT	WT	WT	WT

40	WT	WT	WT	WT	WT	WT

41	WT	WT	WT	WT	WT	WT

42	WT	WT	WT	**F1059L**	WT	WT

43	WT	WT	WT	WT	**V600E**	WT

44	WT	**I24F**	WT	WT	WT	WT

45	**A864T**	WT	WT	WT	WT	WT

46	WT	WT	WT	WT	WT	WT

47	WT	WT	WT	WT	WT	WT

48	WT	WT	WT	WT	WT	WT

49	WT	WT	WT	WT	WT	WT

WT: wild type.

No mutations in HER2 TK domain were detected; 40.5% of samples displayed a silent point mutation at codon 902 (CAG to CAA), both in homo/hemizygous and heterozygous status (data not shown).

The mutational analysis of the EGFR/HER2 intracellular effectors identified mutations in K-RAS, PI3K, B-RAF and PTEN (table 4). Five hotspot mutations in the helical and catalytic domain of PI3K were found in 4/49 specimens (8.2%): two were in codon 545 (ID 763 and ID 12458) and 1 in each of codons 546 (already described as substituted with other non synonymous codons, ID 766, ID 767, ID 6147, and ID 12459), 1047 (ID 775), and 1059. Three samples (6.1%) had point mutations of K-RAS (2 were the previously described G13 D substitution, ID 532, the third was a novel mutation, I24F) and 4 (8.2%) had the V600E mutation (ID 476) of B-RAF. Exons 5, 6, 7, and 8 of PTEN have been sequenced and 3 mutations were found in 2 samples. Namely, sample 23 had Thr to Ile substitution at codon 202 of exon 6 and Glu to Gly substitution at codon 235 of the exon 7; sample 29 had Phe to Leu substitution at codon 271 of exon 8. All PTEN mutations involved codons previously found in other tumors as bearing single base deletions or mutations (ID 5856, ID 5292 and ID 5821).

In 4 cases, mutations of multiple transducers were present simultaneously: sample 22 had mutated B-RAF and K-RAS; sample 23 had mutated K-RAS, PI3K and PTEN; sample 29 had mutated EGFR, PI3K and PTEN; sample 31 had mutated EGFR, PI3K and B-RAF (table 4).

The percentage of PTEN labeled nuclei in tumor samples with activating EGFR or PI3K mutations was higher than in tissues with EGFR and PI3K wild type displaying 62%+31 (score 3+) *vs *39%+26 (score 2+) respectively (p = 0.032). In particular, in the cases with activating mutations involving EGFR and/or PI3K and not PTEN, the mean of PTEN^+ ^cells was 80+19 (score 3+) (p = 0.002 *vs *wild type), suggesting that a compensatory change in the level of the phosphatase might counteract EGFR pathway activation. In agreement, the sample 39 harboring the EGFR stop codon mutation and, presumably, associated with an inactive pathway, had low PTEN expression (9% or score 0).

### Correlation between clinical pathological parameters and biomarkers in BTCs

To test the association between histotype and the analysed biomarkers, a generalization of the Fisher exact test was applied. No association was found between histotype and the presence of EGFR mutations, whereas a highly significant association (p = 0.00004) was detected between histotype and EGFR expression (high in ICCs and ECCs and low in GBCs). A slightly significant association was found between cholongiocarcinoma and p-Akt expression (p = 0.01) and TGF-α expression (0.03). TGF-α and p-MAPK were more expressed in high grade tumors (p = 0.03058 and p = 0.04 respectively). EGFR mutations were more frequently observed in female gender (p = 0.04). The other parameters tested did not give significant associations with histotype.

### Expression of EGFR/HER2 proteins and activation of related transducers in BTC cell lines

**Figure 3:**
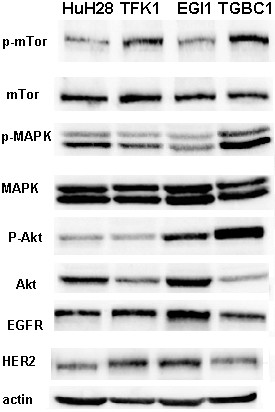
**Western blot analysis of EGFR/HER2 expression and phosphorylation of related signal transducers in BTC cell lines**.

Expression of EGFR and HER2 proteins as well as their molecular pathways were evaluated by western blot analysis on four BTC cell lines. As shown in figure 3, all cell lines expressed EGFR and HER2 receptors. In particular, EGI-1 cell line expressed high levels of EGFR and HER2 proteins and low levels of PTEN. TGBC1-TKB cell line expressed high level of phosphorylated Akt, mTOR and MAPK suggesting sustained activation of these pathways. In addition, HER2 membrane expression was evaluated by immunocitochemistry. ICC cell line HuH28 showed the highest HER2 membrane expression, scored 3+, ECC cell lines EGI-1 and TFK-1 were scored 1+, while GBC cell line TGBC1-TKB showed the lowest HER2 expression (Additional file 1, figure S1).

After 72 h of treatment, everolimus was able to inhibit mTOR phosphorylation in all BTC cell lines, but did not influence Akt and MAPK phosphorylation (Additional file 2, figure S2).

Sorafenib down-regulated MAPK phosphorylation in all cell lines and did not influence mTOR and Akt phosphorylation (Additional file 3, figure S3).

Lapatinib slightly down-regulated Akt phosphorylation in all BTC cell lines, but not MAPK nor mTOR (Additional file 4, figure S4).

Gefitinib down-regulated Akt phosphorylation only in EGI-1 cell line (Additional file 5, figure S5) while erlotinib had no evident effects on Akt/mTOR and MAPK phosphorylation (Additional file 6, figure S6).

### Antiproliferative effect of gemcitabine and EGFR/HER2 pathway inhibitors in BTC cell lines

**Figure 4:**
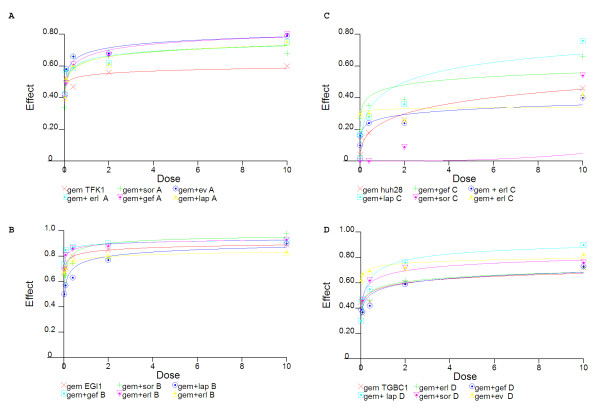
**Dose effect curves of gemcitabine (gem) combined with EGFR/HER2 pathway targeted therapies on TFK1 (A); EGI-1 (B); HuH28(C) and TGBC1-TKB (D)**. After 72 h of treatment with 1:5 scalar doses (10 μM, 2 μM, 400 nM, 80 nM, 16 nM) of gem alone or in combination with targeted therapy at a fixed 1:1 ratio, antiproliferative effect was evaluated using viability test and Chou-Talalay equation by CalcuSyn software as described in the Methods section.

The antiproliferative effect of different molecular targeted drugs blocking EGFR/HER2 receptor or pathways revealed a broad range of response in BTC cell lines (table 5 and figure 4). The ICC cell line HuH28 was resistant to all drugs except lapatinib (Dm = 2.02 μM). Lapatinib also inhibited proliferation of EGI-1 (Dm = 4.02 μM) and TFK1 (Dm = 5.25 μM), while TGBC1-TKB cell line was resistant. EGFR TKIs had a significant effect on ECC cell lines (gefitinib Dm = 1.8 μM in TFK1 and 2.48 μM in EGI-1 whilst erlotinib Dm = 2.59 μM in TFK1 and 5.72 in EGI-1), but no effect was revealed on the GBC cell line TGBC1-TKB. The multi-kinase inhibitor sorafenib had a high efficacy on EGI-1 (Dm = 2.06 μM) and a slight effect on TFK1 and TGBC1-TKB (6.2 μM and 5.9 μM respectively). A reduction of 50% of cell growth was obtained with a relatively low median doses of m-TOR inhibitor everolimus on TFK1 (Dm = 200 nM), on EGI-1 (Dm = 500 nM) and on TGBC1-TKB (Dm = 400 nM).

**Table 5:** Median dose and relative confidential interval values of EGFR/HER2 pathway inhibitors on BTC cell lines.

	TFK1	EGI-1	HuH28	TGBC1-TKB
**Erlotinib Dm (CI)**	2.59 μM	5.72 μM	>10	>10
	(0.17-3.46)	(1.9-7.5)		

**Gefitinib Dm (CI)**	1.8 μM	2.48 μM	>10	>10
	(1.1-2.9)	(1.5-4.03)		

**Lapatinib Dm (CI)**	5.25 μM	4.02 μM	2.02 μM	>10
	(1.01-7.2)	(1.01-5.9)	(1.2-4.62)	

**Sorafenib Dm (CI)**	6.2 μM	2.06 μM	>10	5.9 μM
	(3.1-12.4)	(0.45-9.37)		(3.8-7.9)

**Everolimus Dm (CI)**	0.20 μM	0.5 μM	>10	0.42 μM
	(0.02-1.76)	(0.1-1.5)		(0.33-0.54)

The Median doses (Dm) and relative confidential interval (CI) of targeted therapies which affected 50% proliferation on the four cell lines (TFK1, EGI-1, HuH28, and TGBC1-TKB) were calculated after 72 hrs of drug treatment.

The chemotherapeutic agent gemcitabine was highly efficient on EGI-1 (Dm = 0.74 nM), moderate efficient on TFK-1 and TGBC1-TKB (Dm = 100 nM, Dm = 280 nM respectively) and ineffective on HuH28 (Dm > 10 μM) (table 6).

**Table 6:** Median dose and relative confidential interval values of gemcitabine alone and in combination with EGFR/HER2 pathway inhibitors on BTC cell lines.

Dm μM (confidence interval)	TFK-1	CI*	EGI-1	CI*	HuH28	CI*	TGBC1-TKB	CI*
**Gemcitabine (Gem) **	0.1		0.00074		> 10		0.28	
	(0.003-2.93)		(0.0002-0.002)				(0.12-0.43)	

**Gem + erlotinib**	0.04	0.11	0.00017	0.56	> 10	0.5	0.24	0.8
	(0.03-0.73)		(0.00006-0.0005)				(0.033-2.28)	

**Gem + gefitinib**	0.08	0.15	0.00004	0.15	2.9	0.9	0.26	0.9
	(1.1-2.9)		(0.0000007-0.002)		(0.15-5.3)		(0.13-0.53)	

**Gem + lapatinib**	0.07	0.36	0.03		2.01	1.5	0.13	0.6
	(0.09-29.01)		(0.006-0.17)		(0.46-8.6)		(0.06-0.27)	

**Gem + sorafenib**	0.07	0.017	0.01	0.19	> 10		0.11	0.062
	(0.000009-0.31)		0.00019-1.07)				(0.05-0.23)	

**Gem + everolimus**	0.03	0.15	0.00057	0.015	> 10		0.001	0.07
	0.004-0.43)		(0.00002-0.01)				(0.00007-0.022)	

*CI = combination index indicates synergism when <1

The combination of targeted drugs with gemcitabine allowed a significant reduction of median dose. Interestingly, erlotinib conferred sensitivity to gemcitabine in HuH28, resistant to the same drug as single agent and to all other combinations. In other cell lines, the best result was obtained with the chemotherapeutic agent and everolimus, highly efficient on extrahepatic cell lines, (Dm = 0.13 nM for TFK-1 and Dm = 0.57 nM for EGI-1) and gallbladder cell line (Dm = 1 nM). For the other combinations, responsiveness depended on cell lines.

## Discussion

The increasing of global incidence, poor prognosis and lack of effective therapy make the management of BTCs further emphasize the need of effective therapeutic agents.

Gene status and protein expression of EGFR and HER2 and their pathways may be potential biomarkers for predicting the response to EGFR/HER2 inhibitors.

We observed the presence of 100% EGFR expression in ICCs, 52.6% in ECCs and 38.5% in GBCs.

Mutations in the EGFR TK domain were present in 15% of cases [13]. Furthermore, the incidence of K-RAS mutation was particularly low (6.1%). Interestingly, changes involving codon 12, frequently mutated in other tumor types, were not found in our series. Previous studies of K-RAS mutations in cholangiocarcinoma revealed divergent results [31-35]. A higher occurrence of K-RAS mutations was found in Japan and Germany (ranging from 39% to 54%) relative to other areas such as Thailand (from 0% to 8%) in which this tumor occurred with high frequency. Geographical differences in etiology or carcinogenesis of BTCs might explain this variability.

We observed a lower incidence of B-RAF mutations compared to that reported by Tannapfel and coworkers (8% vs. 21% respectively) [36].

We identified PI3K mutations in 4 cases (8.2%) and PTEN mutations in 2 cases (4.1%).

Multiple mutations of EGFR transducers were observed in some samples. Namely, a total of 14 mutations were found in 8 tumor samples and only 3 samples had a single point mutation. Consistent with previous reports [37,38], the K-RAS and EGFR mutations were not present in the same sample but, in contrast with another report [36], K-RAS and B-RAF mutations were simultaneously present in one case. However, due to the genetic heterogeneity of tumor subclones, we cannot exclude that these mutations would be present in two distinct cell populations.

We observed a rare frequency of PTEN mutations and we did not find any loss of PTEN protein expression in comparison with normal cholangiocytes; rather a stronger labeling intensity and a high percentage of labeled cells were significantly present in tumor cells compared to normal counterparts. In particular, samples displaying EGFR pathway activation due to transducer mutations had the highest percentage of PTEN-labeled cells suggesting that a preserved PTEN function might counteract the EGFR downstream pathway activation. HER2 was overexpressed only in a small group of patients, in accordance with the results obtained by others [39] and no mutations on the TK domain were observed.

The inhibition of EGFR/HER2 pathways in BTCs cell lines demonstrated a broad range of response with EGFR TKIs being more efficient on ECC cell lines. In K-RAS-mutated EGI-1 cells the Dm of these drugs was twice the Dm on K-RAS WT TFK1. Furthermore, the presence of PI3K mutation and PTEN deletion in the HuH28 and TGBC1-TKB cells respectively could probably explain the resistance to these treatments [18,20,21]. Sorafenib was more effective in K-RAS-mutated ECC cell line in which the MAPK pathway had a high level of activation [40]. Lapatinib was more effective in ICC cell line which had high levels of HER2 expression.

On the other hand, lapatinib was less effective on ECC cell lines. Furthermore, GBC cell line was considered resistant (Dm >10 μM). In fact, TFK-1 and EGI-1 cell lines expressed low level of HER2 and GBC cell line was negative. These results are consistent with data obtained in breast cancer and pancreatic preclinical models [41-44]. In addition, a phase II study on breast cancer patients revealed that all the responders showed high level of HER2 expression while the HER2 negative patients were non responders [45]. In our study, the dual inhibition of EGFR and HER2 induced by lapatinib was less effective than the treatment with erlotinib and gefitinib in ECC cell lines. Indeed, in a study by Wiedmann et al., the treatment with NVP-AEE788, an EGFR/HER2/VEGFR-2, was more effective than erlotinib and gefitinib [46]. The direct inhibition of VEGFR-2 could be the gain of function of this drug compared to EGFR and HER2 inhibition. In fact, VEGFR-2 was expressed in ECC cell lines [47]. Moreover, VEGF was overexpressed in ICC and ECC samples from patients and regulated metastasis development [48]. The inhibition of VEGFR and EGFR/HER2 signaling with NVP-AEE788 or vandetanib (ZD6474) might be another interesting alternative approach for the management of BTCs.

Everolimus was effective in all tested cell lines but not in HuH28. Nonetheless, everolimus inhibited the phosphorylation of mTOR in all cell lines. It seems reasonable that in HuH28 a mechanism of resistance that overcomes mTOR inhibition may be active.

Moreover, EGFR/HER2 pathway inhibitors had synergistic effect with gemcitabine treatment. The mTOR inhibition gave rise to the strong synergistic effect in combination with gemcitabine in extrahepatic cell lines. Chung et al demonstrated that more than 80% of extrahepatic BTC displayed mTOR activation [49] that correlated with poor prognosis. Interestingly, EGFR inhibitor erlotinib was able to overcome the resistance to gemcitabine in the intrahepatic cell line HuH28; in fact, intrahepatic specimens showed the highest EGFR expression. Surprisingly, this result was not obtained with gefitinib.

Deepening this study, by gene expression profiling of the cell lines will contribute to the comprehension of the different mechanisms involved in drug response.

## Conclusions

In conclusion, our preclinical results demonstrated that blocking EGFR/HER2 signaling resulted in considerable antiproliferative effects in *in vitro *models of BTC. The employment of targeted therapies may be useful in cholangiocarcinoma treatment and the analysis of EGFR/HER2 pathways in patients could orientate clinicians to the identification of appropriate therapeutic approach.

## Competing interests

The authors declare that they have no competing interests.

## Authors' contributions

YP: designed the study, carried out the experiments, and drafted the manuscript; IS and MR: performed and analysed the immunohistochemical data and critically revised the manuscript; CPN: performed in vitro experiments, mutational analysis and drafted the manuscript; GC: supervised the study and supported with data interpretation; LC performed FISH analysis; GM and JYP performed mutational analysis; GC^3^: conducted statistical data analysis; MA, AB: participated in design and coordination of the study; FL conceived the study.

All authors read and approved the final manuscript.

## Pre-publication history

The pre-publication history for this paper can be accessed here:

http://www.biomedcentral.com/1471-2407/10/631/prepub

## Supplementary Material

Additional file 1 F**igure S1: HER2 expression in BTC cell lines**. A) HuH28, scored 3+, B) EGI-1, scored 1+, C) TFK-1,scored 1+, D) TGBC1-TKB, HER2 negative Click here for file

Additional file 2 F**igure S2: Western blot analysis on mTOR, Akt, MAPK phosphorylation after 72 h treatment with everolimus on BTC cell lines**. Click here for file

Additional file 3 F**igure S3: Western blot analysis on mTOR, Akt, MAPK phosphorylation after 72 h treatment with sorafenib on BTC cell lines**. Click here for file

Additional file 4 F**igure S4: Western blot analysis on mTOR, Akt, MAPK phosphorylation after 72 h treatment with lapatinib on BTC cell lines**. Click here for file

Additional file 5 F**igure S5: Western blot analysis on mTOR, Akt, MAPK phosphorylation after 72 h treatment with gefitinib on BTC cell lines**. Click here for file

Additional file 6 F**igure S6: Western blot analysis on mTOR, Akt, MAPK phosphorylation after 72 h treatment with erlotinib on BTC cell lines**. Click here for file
