# Supplementary figures and images for: Targeting EGFR/HER2 pathways enhances the antiproliferative effect of gemcitabine in biliary tract and gallbladder carcinomas
Source: BMC Cancer. 2010 Nov 18;10:631. doi: 10.1186/1471-2407-10-631 (PMC3000850; doi:10.1186/1471-2407-10-631)

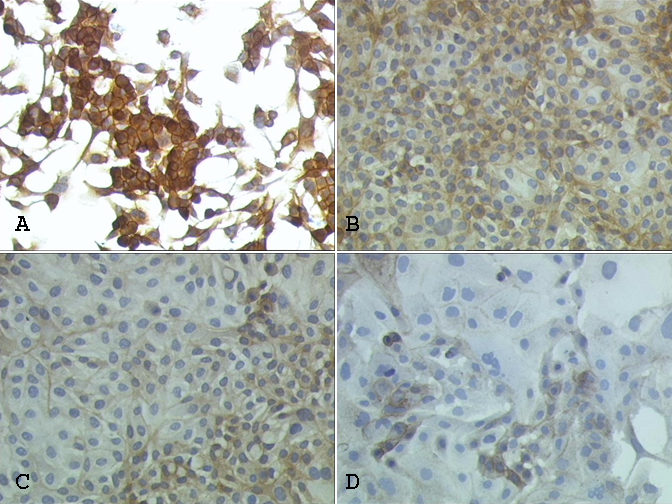

Supplement: Additional file 1 — Figure S1: HER2 expression in BTC cell lines. A) HuH28, scored 3+, B) EGI-1, scored 1+, C) TFK-1,scored 1+, D) TGBC1-TKB, HER2 negative [file 1471-2407-10-631-S1.TIFF]

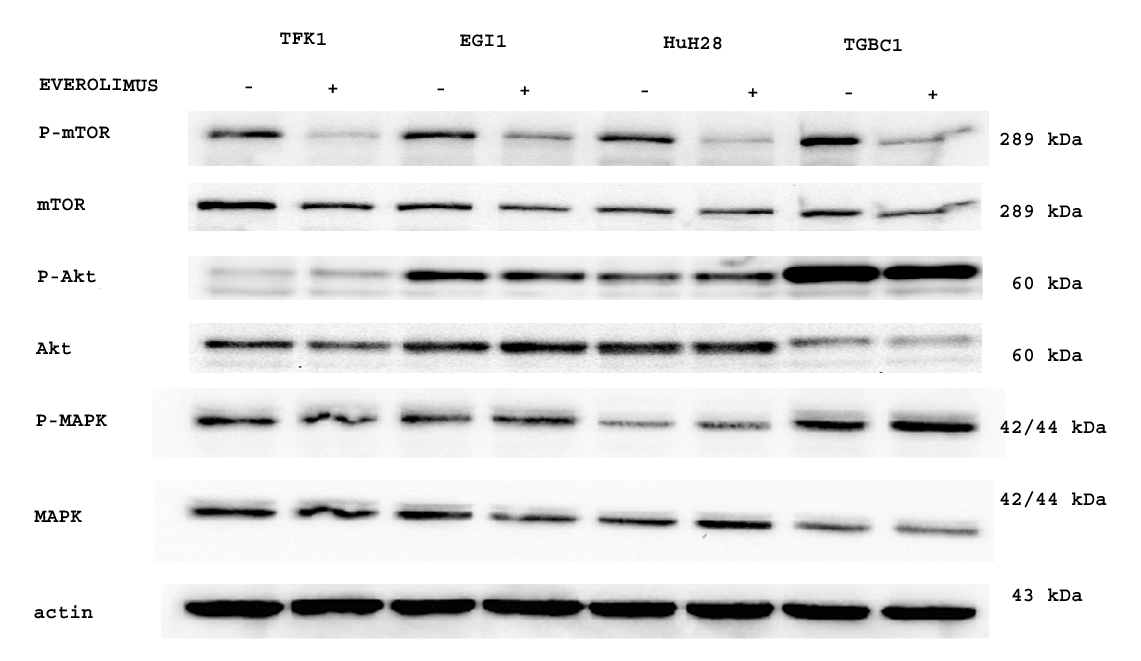

Supplement: Additional file 2 — Figure S2: Western blot analysis on mTOR, Akt, MAPK phosphorylation after 72 h treatment with everolimus on BTC cell lines. [file 1471-2407-10-631-S2.TIFF]

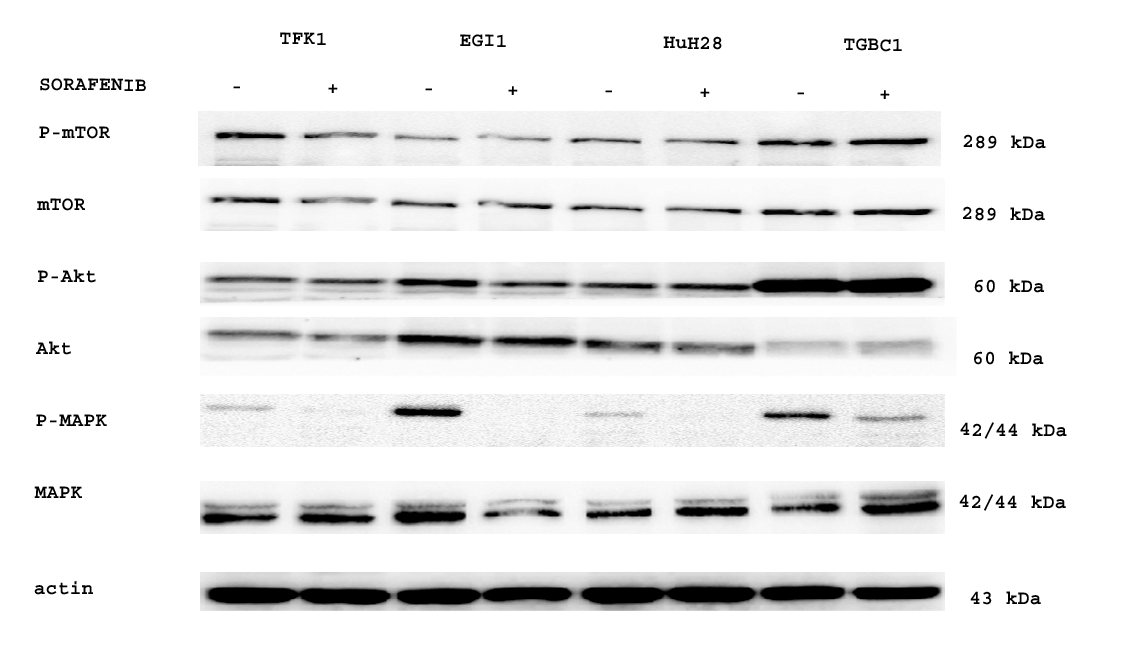

Supplement: Additional file 3 — Figure S3: Western blot analysis on mTOR, Akt, MAPK phosphorylation after 72 h treatment with sorafenib on BTC cell lines. [file 1471-2407-10-631-S3.TIFF]

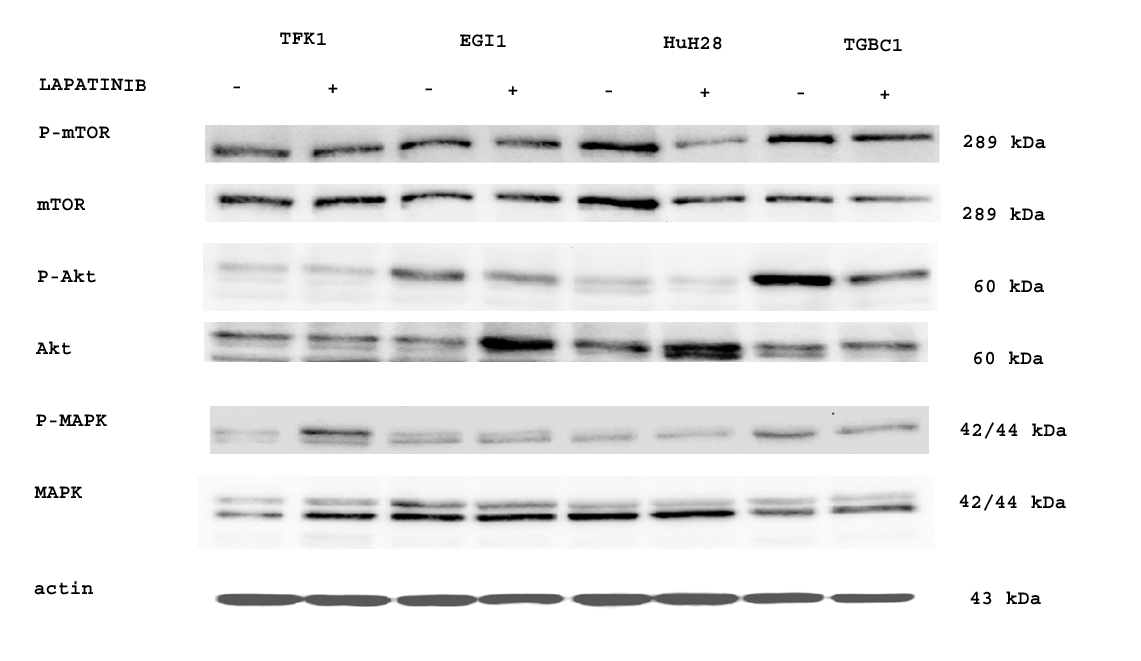

Supplement: Additional file 4 — Figure S4: Western blot analysis on mTOR, Akt, MAPK phosphorylation after 72 h treatment with lapatinib on BTC cell lines. [file 1471-2407-10-631-S4.TIFF]

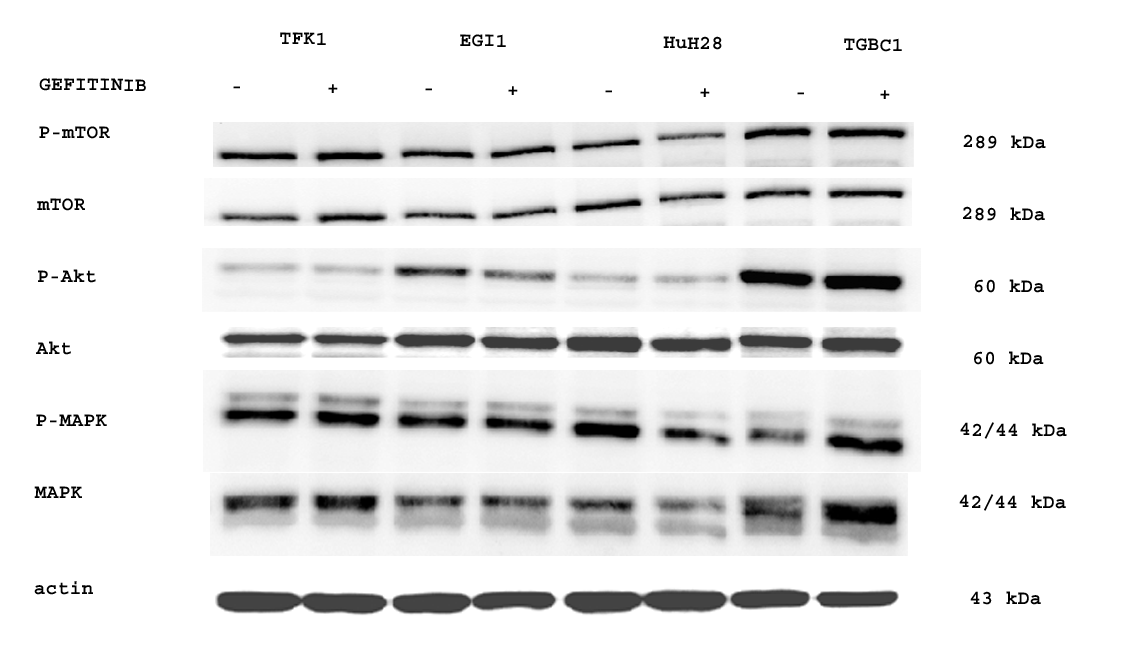

Supplement: Additional file 5 — Figure S5: Western blot analysis on mTOR, Akt, MAPK phosphorylation after 72 h treatment with gefitinib on BTC cell lines. [file 1471-2407-10-631-S5.TIFF]

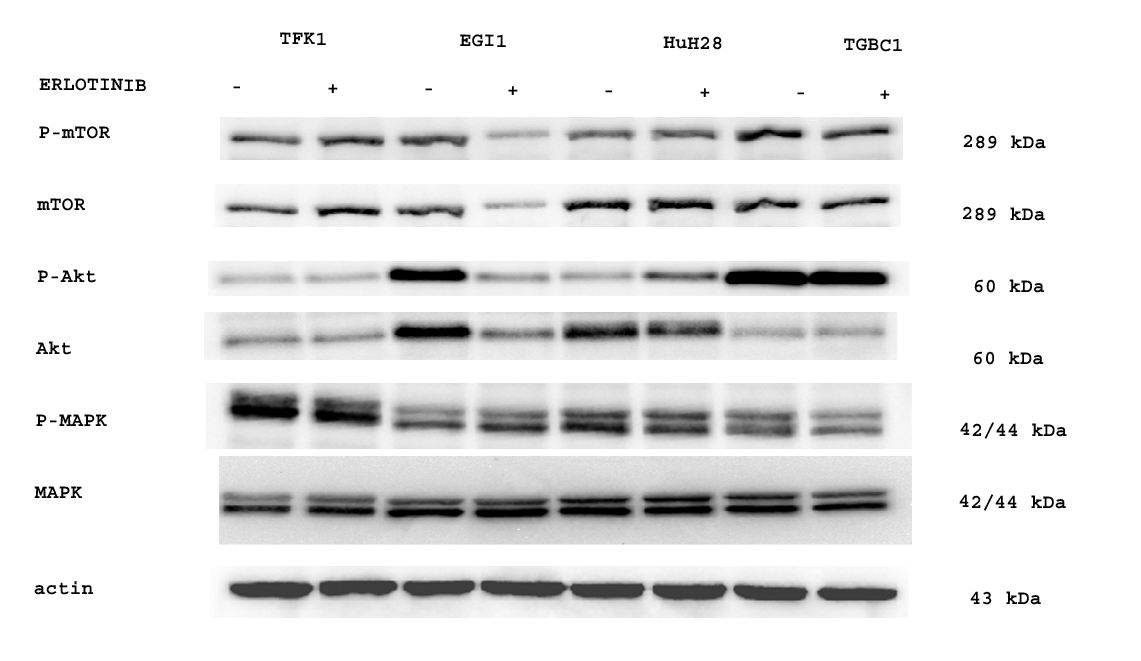

Supplement: Additional file 6 — Figure S6: Western blot analysis on mTOR, Akt, MAPK phosphorylation after 72 h treatment with erlotinib on BTC cell lines. [file 1471-2407-10-631-S6.TIFF]
